# Dr. Joseph Carne Ross

**Published:** 1911-04-22

**Authors:** 


After a few days' illness, Dr. Joseph Carne Ross, late 73rd Perthshire Regiment, 2nd Battalion Black Watch, has died, aged sixty-six. Dr. Ross was an M.D. and F.R.C.P. of Edinburgh, and a Fellow also of the Geographical, Historical, Scottish Antiquarian Societies. He had been physician to the Ancoats Hospital, to the West Cornwall Infirmary, and non-resident house-physician at the Edinburgh Royal Infirmary. He was also the author of "Treatment of Pleurisy with Effusion."

